# Within-Host Microevolution of *Pseudomonas aeruginosa* Urinary Isolates: A Seven-Patient Longitudinal Genomic and Phenotypic Study

**DOI:** 10.3389/fmicb.2020.611246

**Published:** 2021-01-14

**Authors:** Agnès Cottalorda, Marie Leoz, Sandrine Dahyot, François Gravey, Maxime Grand, Thomas Froidure, Fabien Aujoulat, Simon Le Hello, Estelle Jumas-Bilak, Martine Pestel-Caron

**Affiliations:** ^1^Normandie Université, UNIROUEN, Groupe de Recherche sur l’Adapatation Microbienne (GRAM 2.0 EA2656), Rouen, France; ^2^Department of Bacteriology, Rouen University Hospital, Normandie Université, UNIROUEN, Groupe de Recherche sur l’Adapatation Microbienne (GRAM 2.0 EA2656), Rouen, France; ^3^Normandie Université, UNICAEN, Groupe de Recherche sur l’Adapatation Microbienne (GRAM 2.0 EA2656), Caen, France; ^4^Team Pathogènes Hydriques Santé Environnement, UMR 5569 HydroSciences Montpellier, Unité de Bactériologie, UFR Pharmacie, University of Montpellier, Montpellier, France

**Keywords:** *Pseudomonas aeruginosa*, urinary isolates, whole genome sequencing, within-host evolution, single nucleotide polymorphism, large genomic deletion

## Abstract

**Background:**

*Pseudomonas aeruginosa* is responsible for up to 10% of healthcare associated urinary tract infections (UTI), which can be difficult to treat and can lead to bacterial persistence. While numerous whole genome sequencing (WGS) analyses have explored within-host genomic adaptation and microevolution of *P. aeruginosa* during cystic fibrosis (CF) infections, little is known about *P. aeruginosa* adaptation to the urinary tract.

**Results:**

Whole genome sequencing was performed on 108 *P. aeruginosa* urinary isolates, representing up to five isolates collected from 2 to 5 successive urine samples from seven patients hospitalized in a French hospital over 48–488 days. Clone type single nucleotide polymorphisms (ctSNPs) analysis revealed that each patient was colonized by a single clone type (<6000 SNPs between two isolates) at a given time and over time. However, 0–126 SNPs/genome/year were detected over time. Furthermore, large genomic deletions (1–5% of the genome) were identified in late isolates from three patients. For 2 of them, a convergent deletion of 70 genes was observed. Genomic adaptation (SNPs and deletion) occurred preferentially in genes encoding transcriptional regulators, two-component systems, and carbon compound catabolism. This genomic adaptation was significantly associated with a reduced fitness, particularly in artificial urine medium, but no strict correlation was identified between genomic adaptation and biofilm formation.

**Conclusion:**

This study provides the first insight into *P. aeruginosa* within-host evolution in the urinary tract. It was driven by mutational mechanisms and genomic deletions and could lead to phenotypic changes in terms of fitness and biofilm production. Further metabolomic and phenotypic analyses are needed to describe in-depth genotype-phenotype associations in this complex and dynamic host-environment.

## Introduction

*Pseudomonas aeruginosa* is an opportunistic Gram negative pathogen, responsible for 7–10% of healthcare associated urinary tract infections (UTIs) (Lamas Ferreiro et al., 2017). These UTIs preferentially occur in patients with underlying conditions, including patients with indwelling urinary catheter (Djordjevic et al., 2013; Gomila et al., 2018). *P. aeruginosa* can adhere and form biofilm on such surfaces (Olivares et al., 2019), which limits antibiotic diffusion and promotes emergence of antimicrobial resistant strains (Olivares et al., 2019; Soares et al., 2020). Moreover, this allows the microorganism to elude the immune system and persist causing frequent relapses (Gomila et al., 2018). Thus, readmissions are more frequent when UTIs are caused by *P. aeruginosa* (Gomila et al., 2018).

Advances in whole genome sequencing (WGS) techniques have made it possible to explore within-host genomic adaptation and microevolution of *P. aeruginosa*, particularly during cystic fibrosis (CF) infections (Marvig et al., 2015a; Bartell et al., 2019; La Rosa et al., 2019). Within-host evolution in CF patients is driven by three evolutionary modes, including general or directed diversity (Bartell et al., 2019), and convergent molecular evolution (Marvig et al., 2015b; Bartell et al., 2019). For example, the whole genome analysis of 474 longitudinal *P. aeruginosa* isolates collected from 34 CF patients led to the identification of a convergent evolution of 52 genes (Marvig et al., 2015b). These genes were involved in regulatory networks, central metabolism, antimicrobial resistance and secretion of virulence factors. Adaptive trajectories can also result in loss events which were described to be more frequent than gene acquisition (Rau et al., 2012; Bianconi et al., 2018) and could be characterized by large genomic deletions (Rau et al., 2012; Cabot et al., 2016; Hocquet et al., 2016).

Even though identification of genotype-phenotype links can be difficult for complex traits governed by multiple regulatory networks (Bartell et al., 2019), phenotypic changes have been previously described during persistent CF infections. They included a decreased secretion of virulence factors, the emergence of hypermutable strains often associated with an increased antimicrobial resistance, a switch from planktonic to sessile lifestyle, or a phenotypic conversion to mucoid or rugose small colony variants (Cabot et al., 2016; Hocquet et al., 2016; Winstanley et al., 2016; Klockgether and Tümmler, 2017; Pestrak et al., 2018; Colque et al., 2020).

In contrast, no study has explored to date *P. aeruginosa* within-host genomic and phenotypic evolution in the urinary tract. Indeed, most of the whole genome sequences of *P. aeruginosa* urinary isolates currently consist of genome announcements (Tielen et al., 2014; Wibberg et al., 2014, 2015; Vorhölter et al., 2015; Hussain et al., 2017).

In this context, we sequenced the whole genome of sequential urinary isolates of seven patients colonized and/or infected by *P. aeruginosa* to analyze the nature and extent of their genomic changes and provide an overview of within-host *P. aeruginosa* diversity at a given time and over time. We have also explored the impact of genomic changes on phenotypic traits such as fitness and biofilm formation.

## Materials and Methods

### Bacterial Isolates and Clinical Data

**FIGURE 1:**
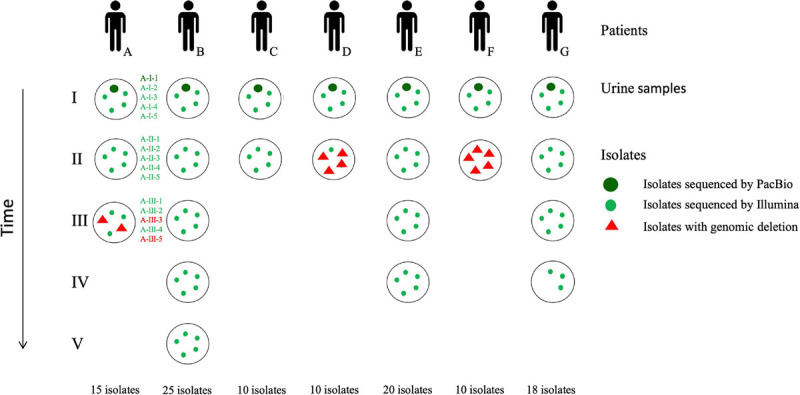
Flowchart of the *P. aeruginosa* collection studied. Patients (A–G) are illustrated by black figures. Urine cultures (roman numbers) collected from each patient are represented by black circles. Up to five isolates (red or green dots; arabic numbers) were collected from each urine culture. Isolates with genomic deletions are indicated by red triangles. Only three isolates were collected from the last urine sample for patient G because of a low bacteriuria. As an example, nomenclature of the isolates collected from each urine sample is represented for patient A.

Two to five monomicrobial urine cultures (named I–V) of *P. aeruginosa* (regardless of the level of leucocyturia) were prospectively and consecutively collected from seven patients (A–G) over a 27-month period (from June 2016 to August 2018), with up to five isolates per sample (1–5), representing a total of 108 isolates (Table 1 and Figure 1). Isolates from the first sample collected were considered as early isolates while those from samples obtained at the subsequent times were considered as late isolates. Clinical data were collected from computerized medical and biological records of the hospital in parallel with the collection of the urinary isolates. According to the French regulation on observational database analyses, this study did not need specific informed consent requirements; it was registered by the Directorate of Clinical Research and Innovation of the Rouen University Hospital Centre under the number 2018/413/OB. The patients had episodes of UTI and/or asymptomatic bacteriuria (AB) averaging 192 days apart (ranging from 48 to 488 days).

**TABLE 1:** Clinical context associated with the 108 *P. aeruginosa* urinary isolates studied.

**Patient**		**A**	**B**	**C**	**D**	**E**	**F**	**G**
Age range (years)		70–74	65–69	80–84	65–69	60–64	15–19	75–79
Urinary comorbidity^*a*^		N	Y	Y	Y	Y	Y	Y
Immunocompromised conditions^*b*^		Y	Y	N	Y	Y	N	N
**Clinical context [time]^*c*^**								
	Urine sample I	**UTI** [D_0_]	AB* [D_0_]	UTI* [D_0_]	AB [D_0_]	UTI* [D_0_]	AB* [D_0_]	UTI* [D_0_]
	Urine sample II	**UTI** [D_14_]	AB* [D_33_]	UTI* [D_48_]	AB [D_259_]	AB [D_28_]	UTI* [D_113_]	AB* [D_46_]
	Urine sample III	AB [D_82_]	AB* [D_296_]			AB [D_70_]		AB* [D_90_]
	Urine sample IV		AB* [D_362_]			AB [D_81_]		UTI* [D_271_]
	Urine sample V		AB* [D_488_]					

Letters (A–G) identify patients. Roman numerals (I–V) correspond to urine sample numbers in the series collected from a given patient. ^*a*^Urinary comorbidity includes neurogenic bladder, benign prostate hypertrophy, urinary tract diversion, and renal transplant. ^*b*^Immunocompromised conditions include immunodepression (such as AIDS, lymphoma, leukemia) or immunosuppressive treatment. ^*c*^Days (D) between a given urine sample and the first one. *Indicates urinary catheterization. Y, yes; N, no; UTI, urinary tract infection; AB, asymptomatic bacteriuria.

Bacterial isolates were identified by matrix-assisted laser desorption/ionization time-of-flight (MALDI-TOF) mass spectrometry (Bruker Daltonik GmbH, Bremen, Germany) and stored at −80°C for further analyses.

### Antibiotic Susceptibility Testing

Antibiotic susceptibility testing (AST) was performed for all isolates by disk diffusion method on Mueller-Hinton agar (Bio-Rad^®^, Marnes-la-Coquette, France) according to the criteria of the European Committee on Antimicrobial Susceptibility Testing^1^. Antibiotic disks were purchased from Bio-Rad^®^. Consensus recommendations (Magiorakos et al., 2012) were used to evaluate the proportion of Multi Drug-Resistant (MDR, not susceptible to at least three antimicrobial groups) and extensively Drug Resistant (XDR, not susceptible to at least six antimicrobial groups) isolates, considering the following six antimicrobial groups: penicillin (ticarcillin, ticarcillin-clavulanate, piperacillin, piperacillin-tazobactam), cephalosporins (ceftazidime, cefepime), monobactams (aztreonam), carbapenems (imipenem, meropenem), aminoglycosides (gentamicin, tobramycin, amikacin), and fluoroquinolones (ciprofloxacin, levofloxacin). The other isolates – resistant to none, one or two classes of antipseudomonal agents – were defined as non-MDR. The *P. aeruginosa* strain ATCC 27853 was used as a quality control strain in each experiment.

Of note in a parallel study (Cottalorda et al., unpublished), the antimicrobial resistance profiles, identified for isolates collected from the first urine sample, were also explored for these seven patients.

### DNA Extraction, Library Construction, Genome Sequencing, and *De novo* Assembly

#### PacBio Library Construction and Sequencing

For each patient, the first sample isolate with the higher rate of antimicrobial resistance (named X-I-1 where X was a capital letter from A to G) was sequenced with the Pacific Biosciences (PacBio) technology. It was considered as the intra-patient reference sequence. Genomic DNA was extracted using the NucleoSpin Microbial DNA kit (Macherey-Nagel, Illkirch-Graffenstaden, France) according to the manufacturer’s recommendations. The SMRTbell library was prepared using a SMRTbell Express 2 Template prep kit (Pacific Biosciences^®^, California Inc., United States), following the “procedure and checklist preparing Multiplexed Microbial Libraries using SMRTbell Express Template prep kit 2.0” protocol. Single-molecule Real-time long reads sequencing was performed at the Gentyane Sequencing Platform (Clermont-Ferrand, France) with a PacBio Sequel Sequencer (Pacific Biosciences^®^, Menlo Park, CA, United States). *De novo* assembly was performed using “Microbial Assembly” pipeline in PacBio SMRT Link v8.0.0.80529, using default parameters, except “Genome Size,” which was set to X.

#### Illumina NextSeq 500 Library Construction

The genome of the 101 remaining isolates was sequenced by the “Plateforme de Microbiologie Mutualisée P2M” (Institut Pasteur, Paris, France) using the MagNA Pure 96 Instrument (Roche Diagnostics, Meylan, France) for genomic DNA extraction, and the Nextera XT library kit (Illumina Inc.), the NextSeq 500 sequencing system (Illumina Inc.). Paired-end reads were submitted to pre-processing using fqCleaner and to *de novo* assembly using SPADES, with a 50X minimum average sequencing depth.

### Genome Annotation, Genotyping, Clone Type Definition

Genome annotation was performed for the seven isolates sequenced by PacBio using the Prokaryotic Genome Annotation Pipeline (PGAP) version 2019-08-22 (Tatusova et al., 2016)^2^.

*In silico* Multilocus Sequence Typing (MLST) genotyping was performed according to the scheme established by Curran et al. (2004), using KMA software version 1.2.12 (Clausen et al., 2018) with default settings^3^, and the online database (updated on 2019-03-18) of the Center for Genomic Epidemiology (Larsen et al., 2012).

Phylogenetic analysis of the 108 Illumina and PacBio assemblies produced by SPADES was performed using Parsnp software version 1.2 (Treangen et al., 2014) with default parameters using the A-I-1 strain as reference. The iTOL software (Letunic and Bork, 2019) was used to visualize and edit the phylogenetic tree and identify phylogenetic clusters (bootstrap of 100) inside the *P. aeruginosa* urinary population.

Illumina reads were aligned against every PacBio reference sequences using Snippy software version 4.4.0 (available on https://github.com/tseemann/snippy/). Clone type SNPs (ctSNPs), bounded by at least 50 exact base-pair matches on both sides, were extracted from the alignments using a custom script^4^. Isolates were considered to belong to different clone types when more than 6 000 ctSNPs were found between their genomes (Marvig et al., 2015b).

PlasmidFinder 2.1, available on the Center for Genomic Epidemiology database^5^ was used to identify plasmid in genome sequences.

### SNPs Analysis

An intra-patient SNPs analysis was performed using Snippy software, by mapping Illumina reads to their corresponding PacBio reference sequence. This intra-patient mapping approach was chosen over the fast Parsnp analysis of genome assemblies (well fit for inter-patient scaled phylogenies) because it allowed a fine-scale analysis of the SNPs from raw reads, and maximized the size of the intra-patient core genomes. SNPs analysis was manually curated. When SNPs were found in all the isolates sequenced by Illumina, they were considered specific to their common PacBio reference sequence even though we cannot exclude that some were in fact artifacts due to the use of distinct sequencing approaches (PacBio vs. Illumina). Snippy called the SnpEff software (Cingolani et al., 2012) to annotate and predict the effect of each mutation. To identify the corresponding PAO1 locus tags, gene sequences from each PacBio reference genome were blasted against all PAO1 gene sequences retrieved from https://www.pseudomonas.com/. Gene sequences that with no blast hit against PAO1 were classified as “hypothetical, unclassified, unknown.” Genes where SNPs occurred were classified into functional categories using PseudoCAP available on the *Pseudomonas* Genome Database^6^. SNPs were classified as low (e.g., synonymous mutations), moderate (e.g., missense mutations), and high effect (e.g., stop gained). SNPs that were not identified in early isolates (collected from the first urine sample for each patient) but present in at least two of late isolates were further described as “late stage SNPs.”

The core alignments produced by Snippy software for each patient were used to infer within-host phylogenies: Neighbor Joining trees were produced using MEGA version X (Kumar et al., 2018), with a Tamura Nei evolutionary model, rate heterogeneity following a Gamma distribution (parameter = 1), and complete deletion of gaps and missing data.

### Characterization of Genomic Deletions

Genomic deletions were visualized using CGView software (Stothard and Wishart, 2005), based on the core alignments produced by Snippy. Deletions were also identified using KMA software version 1.2.12 (see text footnote 3), with Illumina reads as query and the CDS identified by pgap from their corresponding PacBio reference genome as database. The proportion of the core and accessory genome were calculated based on the core alignments produced by Snippy. To identify the corresponding PAO1 locus tags, gene sequences from each PacBio reference genome were blasted against all PAO1 gene sequences retrieved from https://www.pseudomonas.com/. Gene sequences that with no blast hit against PAO1 were classified as “hypothetical, unclassified, unknown.” The deleted genes were classified into functional categories using PseudoCAP available on the *Pseudomonas* Genome Database (see text footnote 6).

Genomic deletions were confirmed by PCR amplification. Briefly, genomic DNA was extracted using the InstaGene^TM^ Matrix kit (Bio-Rad) following the manufacturer’s recommendations. PCRs were then performed on a Veriti Thermal Cycler (Applied Biosystems, Foster City, CA, United States) in a final volume of 25 μL containing 12.5 μL GoTaq^®^ Green Master Mix (Promega, Charbonnières-les-Bains, France), 0.50 mM of each primer (Supplementary Table 1) and 5 μL of DNA. In each case, three primer pairs were used: two that bordered each side of the potentially deleted fragment and allowed the amplification for isolates without genomic deletion, and one outside the deleted region, which allowed the amplification for isolates with genomic deletion. Two genomes without deletion [the intra-patient reference (X-I-1) and when possible, an isolate collected from the last urine sample] obtained from the same patient were used as a control.

### Biofilm Formation Assay

Biofilm formation was assessed by a crystal violet staining assay as previously described (O’Toole, 2011) with minor modifications. Briefly, strains were grown overnight in trypticase soya broth (Thermo Fisher Scientific, Les Ulis, France) supplemented with 1% glucose (w/v) (VWR, Leuven, Belgium) (Brooks and Keevil, 1997). Overnight cultures were adjusted to an optical density at 600_*nm*_ (OD_600__*nm*_) of 0.01. In a 96-well plastic microtiter plate (Thermo Fisher Scientific, Illkirch-Graffenstaden, France), 100 μL of the standardized samples were inoculated per well in triplicate. After 24 h of static incubation at 37°C, supernatants were removed, and the cells that remained attached to the microtiter wells were washed twice with water by submersion. Cells were stained with 125 μL of an aqueous solution of 0.1% crystal violet. After 15 min of incubation at room temperature, the plate was rinsed thrice with water by submersion and dried for 2 h. Cells were washed and resuspended in 50% (v/v) ethanol. OD_590__*nm*_ of the resuspended biofilm was determined and normalized to the OD_600__*nm*_ of the corresponding planktonic cells. All experiments were performed at least three times. The *P. aeruginosa* strain PA14 was used as a positive control and fresh medium as a negative control for each experiment. All data were expressed as percent of biofilm formation relative to the positive control. Isolates forming less than 50% of biofilm compared to PA14 were considered as low biofilm-producers, whereas isolates forming more than 50% were considered as high biofilm-producers. Statistical analyses were performed by Kruskal–Wallis test on R^©^ software (v.3.5.1).

### Fitness Assays

Growth curves were performed in two distinct media: trypticase soya broth, and artificial urine medium (AUM) according to Brooks and Keevil’ protocol (Brooks and Keevil, 1997) with minor modifications. Briefly, 2-(*N*-morpholino)ethanesulfonic acid (MES buffer) at 100 mM was added to the previously described AUM in order to limit precipitates formation. Overnight cultures were adjusted to an OD_600__*nm*_ of 0.01. In a 96-well plastic microtiter plate, 200 μL of standardized samples were inoculated per well in triplicate. The plate was placed inside a humidity cassette and incubated at 37°C for 24 h under continuous double-orbital shaking (108 rpm) into the *Spark*^®^ multimode microplate reader (Tecan, Männedorf, Switzerland). OD_600__*nm*_ was measured every 15 min. All experiments were performed at least three times. The *P. aeruginosa* strain PA14 was used as an internal control and fresh medium as a negative control for each experiment. Statistical analyses were performed by Kruskal–Wallis test on R^©^ software (v.3.5.1). The generation time ratio was defined for a given patient as the mean generation time of isolates with genomic deletion on the mean generation time of isolates without one.

## Results

### Clinical Data

During a 27-month period, seven hospitalized patients (A–G) with more than one urine sample positive to *P. aeruginosa* were included in this study (Figure 1).

**FIGURE 2:**
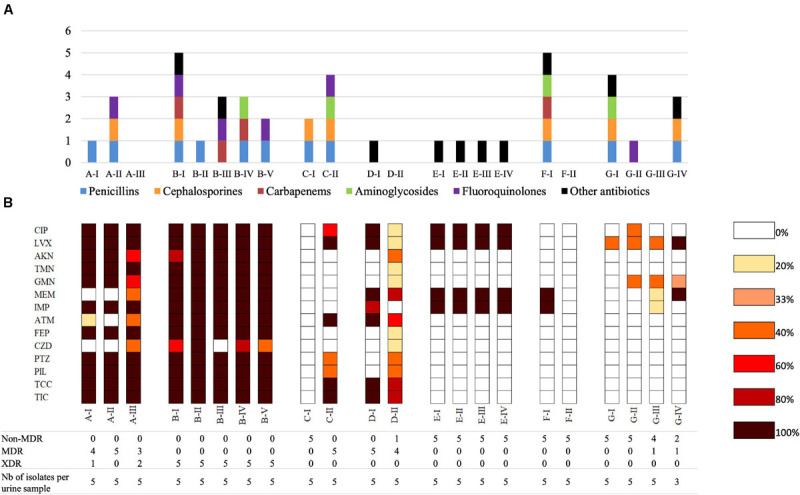
Antimicrobial resistance of *P. aeruginosa* urinary isolates. **(A)** Previous antibiotic therapy received by patients; in the previous 6 months before the first urine culture or in the laps of time between two urine cultures. **(B)** Heatmap representing antibiotic resistance of *P. aeruginosa* urinary isolates per urine culture. For each urine culture, the percentages of isolates resistant to each antibiotic were calculated. For example, when 2 of 5 isolates collected from a given urine sample were resistant to ticarcillin, the resistance rate was 20%. TIC, ticarcillin; TCC, ticarcillin-clavulanate; PIL, piperacillin; PTZ, piperacillin-tazobactam; CZD, ceftazidime; FEP, cefepime; ATM, aztreonam; IMP, imipenem; MEM, meropenem; GMN, gentamicin; TMN, tobramycin; AKN, amikacin; CIP, ciprofloxacin; LVX, levofloxacin. The numbers below the heatmap represent the number of isolates with non-MDR, MDR or XDR resistance profiles within a given urine sample.

Chronic asymptomatic bacteriuria (AB) was observed for two patients (B and D) while chronic UTI was observed for one patient (C). A switch from UTI to AB (patients A and E), from AB to UTI (patient F) or successive switches from UTI and AB (patient G) were identified (Table 1). Of note, four patients (B, C, F, and G) required long term urinary catheterization. Six patients had urinary disorders, including benign prostate hypertrophy (C, D, G), urinary tract diversion (B, F), and kidney transplant (E). All patients received at least one antibiotic treatment during the 6 months before urine collection, and five of them (A, B, C, E, and G) also received antibiotics in the interval of their urine samples (Table 1 and Figure 2A and Supplementary Table 2).

### Antimicrobial Resistance Profiles

Isolates from the first urine sample exhibited a non-MDR phenotype for four patients (C, E, F, G), a MDR phenotype for one patient (D), and a XDR phenotype for one patient (B). Within-sample diversity was observed between isolates collected from patient A (four MDR-isolates and one XDR) (Figure 2B).

Over time, the rate of antimicrobial resistance was similar for isolates of two patients (B, E) while it decreased for patient F, and increased for patients C and G (Figure 2B). Within-sample diversity of antimicrobial resistance profiles increased over time for three patients (A, D, G) (Figure 2B and Supplementary Table 3). No plasmid that could explain the acquisition of these resistance profiles have been identified in the genome sequences.

Surprisingly, no strict association was identified between antimicrobial resistance (Figure 2B) and previous antibiotic therapy (Figure 2A). Indeed, resistance to a given group of antibiotics was not systematically associated with previous antimicrobial therapy by one molecule of that group, suggesting acquisition of cross-resistance. Similarly, the increase of antimicrobial resistance of late isolates was not completely explained by antibiotic treatments during the period between urine samples.

### Genotyping and Phylogeny of *P. aeruginosa* Urinary Population

Sequencing data are presented in Supplementary Table 4. The number of predicted genes per genome sequenced by PacBio and annotated with PGAP ranged from 5,991 to 6,886.

*In silico* MLST analysis performed for the 108 urinary isolates revealed six sequence types (STs): ST111, ST232, ST235, ST308, ST633, and ST3227. All isolates from a given patient, within a given sample but also over time, belonged to the same ST. Two patients (D and E) had isolates with the same ST (ST308). The phylogenetic analysis of the 108 core genomes (draft assemblies) led to the identification of six clusters that were congruent with the 6 STs identified (Supplementary Figure 1A).

Clone type single nucleotide polymorphisms analysis led to the identification of six clone types, in accordance with the STs identified. The average number of ctSNPs between isolates from a given patient was seven (from 0 to 123) while it was 26,242 between patients (from 412 to 33,638) (Supplementary Table 5). Of note, ST308 genomes belonged to a single clone type (number of ctSNPs between patients D and E from 412 to 1,212), even though isolates were collected from distinct patients. However, there was no clinical support for a cross infection between patients D and E, since they were hospitalized in separate units at distinct times and were not epidemiologically linked. Sequences sampled from each patient formed two distinct and well supported phylogenetic clusters (Supplementary Figure 1B).

### Within-Host Evolution by SNPs

To characterize over-time evolution of within-host single-clone types, we investigated all SNPs identified for each isolate, depending on the time of urine sampling (Supplementary Table 6). For the seven patients, the mean number of SNPs was higher for the late isolates (8.6 ± 25) than for the isolates collected from the first urine sample (0.8 ± 1). The mutation rate ranged from 0 to 126 SNPs per genome per year (Table 2).

**TABLE 2:** Average number of SNPs per urine sample for the seven patients studied.

**Patient**		**A**	**B**	**C**	**D**	**E**	**F**	**G**
Number of positions investigated in each core genome		6,598,293	6,803,544	6,266,825	6,854,231	6,890,906	6,472,838	6,416,922
Urine culture	I	0.8	1.4	0.2	2	0.6	0	0.4
	II	0.2 [10]	2.8 [31]	1.2 [9]	89.6 [126]	0 [0]	7.4 [24]	0 [0]
	III	0.6 [3]	6.4 [8]			0.4 [2]		0 [0]
	IV		9 [9]			0.2 [1]		2.7 [4]
	V		5.8 [4]					

*Illumina reads were mapped to the corresponding PacBio reference using Snippy software. The total number of positions investigated in each intra-patient core genome is indicated. The mean number of SNPs/year (mutation rate, in square brackets) was calculated for isolates from each urine culture, based on the time elapsed since the previous urine culture.*

**FIGURE 3:**
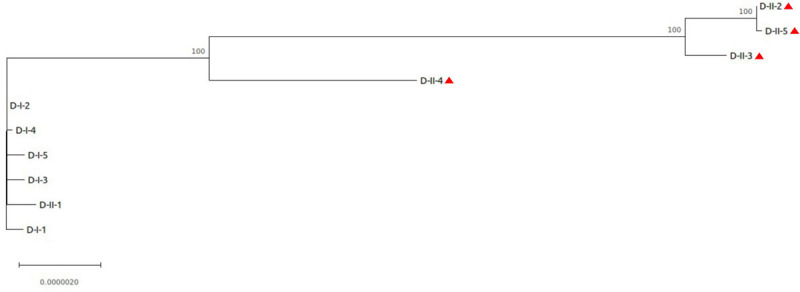
Phylogenetic analysis of the isolates collected from patient D. The core-genome alignment produced by snippy was used for phylogenetic inference using MEGA software. The phylogenetic tree was rooted on the PacBio reference sequence D-I-1. Isolates with genomic deletion are identified by a red triangle. Bootstrap values are indicated when >70. The length of the scale bar represents 0.0000020 substitutions per site, corresponding to 13.71 SNPs in this 6,854,231 nucleotide-long core genome alignment.

D-isolates had the highest number of SNPs, with an average of 2 SNPs per isolate in the first urine sample, increasing to 90 in the second one (Table 2). Phylogenetic analysis of the core-genome sequences sampled from this patient revealed distinct sublineages in the second urine sample, consistent with the high number of SNPs observed (Figure 3). Indeed, one late isolate (D-II-1) was phylogenetically related to the early isolates, and did not share any SNP with the other four late isolates that clustered separately. Interestingly, no mutation was detected in DNA mismatch repair genes (like *mutS* or *mutL*) but 2 SNPs were identified in genes involved in DNA replication, recombination, modification and repair (a missense SNP, Asp559Asn, in *uvrA*, and a nonsense SNP at position 309/530 in the gene homologous of PA1405 at locus tag H7Q90_28210).

To identify genetic mutations that could be involved in phenotypic adaptation, we focused on late stage SNPs (i.e., SNPs that were not identified in early isolates but present in at least two of late isolates) with moderate (non-synonymous mutations) or high (stop gained) effect.

No such SNP was detected in isolates collected from three patients (A, C, and E), while 2–79 late stage SNPs were identified for the other patients (B, D, F, and G). No pathoadaptive genes reflecting convergent evolution by these SNPs were identified for the isolates collected from the four patients. However, SNPs preferentially occurred in genes encoding transcriptional regulators or involved in two component systems (Table 3). However, no pathoadaptive genes reflecting convergent evolution by SNPs was identified between isolates collected from distinct patients.

**TABLE 3:** PseudoCAP function classification of genes affected by late stage SNPs with moderate or high effect identified in isolates collected from four patients (B, D, F, G).

**PseudoCAP manually assigned functions**	**B- isolates**	**D- isolates**	**F- isolates**	**G- isolates**
Adaptation, protection	0%	2%	**11%**	0%
Amino acid biosynthesis and metabolism	0%	2%	0%	0%
Antibiotic resistance and susceptibility	0%	2%	0%	0%
Biosynthesis of cofactors, prosthetic groups and carriers	0%	2%	0%	0%
Carbon compound catabolism	0%	4%	0%	**33%**
Cell division	0%	2%	0%	0%
Cell wall/LPS/capsule	0%	4%	**33%**	0%
Central intermediary metabolism	0%	1%	0%	0%
Chemotaxis	**33%**	1%	0%	0%
DNA replication, recombination, modification and repair	0%	2%	0%	0%
Energy metabolism	0%	1%	0%	0%
Fatty acid and phospholipid metabolism	0%	0%	0%	**33%**
Membrane proteins	0%	**13%**	0%	0%
Motility and Attachment	**33%**	2%	0%	0%
Nucleotide biosynthesis and metabolism	0%	2%	0%	0%
Protein secretion/export apparatus	0%	3%	0%	0%
Putative enzymes	0%	**6%**	0%	0%
Secreted Factors (toxins, enzymes, alginate)	0%	1%	**11%**	0%
Transcription, RNA processing and degradation	0%	3%	0%	0%
Transcriptional regulators	0%	**7%**	**22%**	**33%**
Translation, post-translational modification, degradation	0%	4%	0%	0%
Transport of small molecules	0%	**5%**	0%	0%
Two-component regulatory systems	**33%**	3%	**11%**	0%
Hypothetical, unclassified, unknown	0%	27%	11%	0%

*Percent of SNPs identified per gene function are presented here. PseudoCAP functions are available on the *Pseudomonas* Genome Database (https://www.pseudomonas.com/). When over than 5% of genes for a given PseudoCAP function were affected (except for “Hypothetical, unclassified, unknown” function), values were indicated in bold.*

Single nucleotide polymorphisms were identified in genes involved in virulence for isolates collected from patient D, F, and G. For D-isolates, eight SNPs were identified in genes involved in motility and adherence (*fleS, fimT*), in type III and VI secretion systems [*exsA* (nonsense SNP), *icmF1, hsiG3*], in ferric uptake regulator (FUR) regulon (PA2094 and PA2402), or in stringent response (antitoxin *higA*). For F-isolates, three SNPs were identified in genes involved in alginate biosynthesis or alginate regulation and biofilm formation [*algG, algB*, and *mucA* (nonsense SNP)]. These isolates presented a mucoid morphotype after 48 h of culture (data not shown), which could be explained by *mucA* inactivation (an anti-sigma factor of the *algU* gene involved in alginate biosynthesis). Finally, one SNP was observed in the *fecI* sigma factor of the FUR regulon in G-isolates.

Of note, SNP occurring in genes involved in antimicrobial resistance were only identified for D-isolates (*mexS*).

### Within-Host Evolution by Genomic Deletion

**FIGURE 4:**
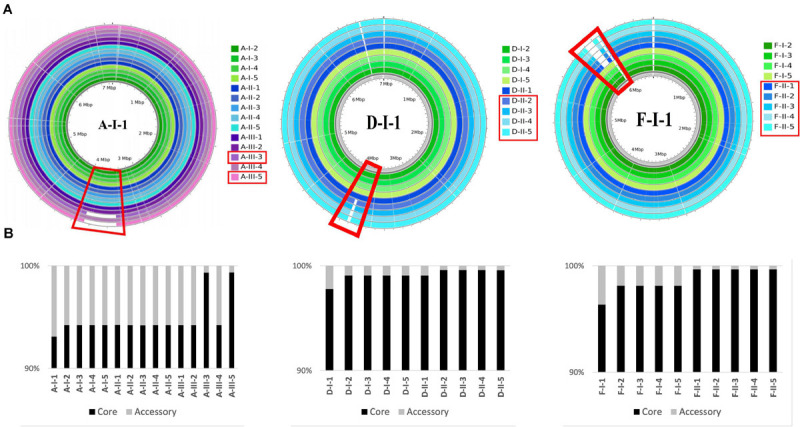
Genomic deletions identified in late isolates collected from three patients (A, D, F). **(A)** Genome coverage for isolates collected from three patients (A, D, F). Genome coverage is represented according to Illumina read mapping by Snippy on their corresponding PacBio reference sequence. Green, blue, and purple colors indicate that the isolates are from sample I, II, and III, respectively. Samples and genomic regions where large deletions occurred in late isolates are framed by red rectangles. **(B)** Proportion of the core and accessory genomes for isolates collected from three patients (A, D, F). The proportions were calculated based on the core alignments produced by Snippy. The size of the core genome was 6,598,293 bp, 6,854,231 bp, and 6,472,838 bp, for A-, D-, and F-isolates, respectively.

For three patients (A, D, and F), at least two of the late isolates had a smaller genome size than isolates collected from the first urine sample (Supplementary Table 7), as shown by the read mapping on the intra-patient reference sequence (Figures 4A,B). Deletions represented up to 5% of the size of the reference genome for A- isolates, and about 1% for D- and F- isolates. All these genomic deletions were experimentally confirmed by PCR (Supplementary Table 1).

The largest genomic deletion (361 kbp) was observed in two late A- isolates (A-III-3 and -5), and led to the loss of 375 coding protein sequences (CDS). Genomic deletions of 35 kbp (48 CDS) and 101 kbp (96 CDS) and were observed for D-II-2 to D-II-5 isolates and F-II-1 to F-II-5 isolates, respectively. The deleted fragment of D- and F-isolates was immediately bordered by direct repeats (DR). For the F-isolates, the 1677 bp DR sequence corresponded to an insertion sequence (IS) that belongs to the IS*1182* transposase family. Interestingly, the same IS was found at seven positions in the F-I-1 genome. Furthermore, the deletions in A- and D-isolates led to the loss of 11 and 19 phage related CDS, respectively.

For A- and F-isolates, 70 deleted CDS were common (homologous to PA1965 to PA2034 genes), suggesting a convergent evolution (Table 4). This region included genes involved in antimicrobial resistance (*mexX/Y/Z* genes), regulatory systems (transcriptional regulators or two component systems), carbon compound catabolism (*liu, exa*), and the genes *hmgA* (encoding a homogentisate 1,2-dioxygenase) and *galU* (encoding an UDP-glucose phosphorylase involved in lipopolysaccharide biosynthesis) (Cabot et al., 2016; López-Causapé et al., 2018; Wardell et al., 2019). Interestingly, isolates with the *hmgA* deletion produced a red pigment (pyomelanin) when they were grown 24 h on TSA (data not shown), as previously reported (Cabot et al., 2016).

**TABLE 4:** PseudoCAP function classification of deleted genes identified in isolates collected from three patients (A, D, F).

**PseudoCAP manually assigned functions**	**A-isolates**	**D-isolates**	**F-isolates**	**A- and F- isolates**
Adaptation, Protection	1%	0%	1%	0%
Amino acid biosynthesis and metabolism	2%	0%	3%	4%
Antibiotic resistance and susceptibility	1%	0%	3%	2%
Biosynthesis of cofactors, prosthetic groups and carriers	2%	0%	**6%**	**8%**
Carbon compound catabolism	**5%**	0%	**13%**	**16%**
Cell wall/LPS/Capsule	0%	0%	1%	0%
Central intermediary metabolism	2%	0%	2%	2%
Chaperones and heat shock proteins	1%	0%	1%	1%
DNA replication, recombination, modification and repair	1%	0%	0%	0%
Energy metabolism	1%	0%	1%	1%
Fatty acid and phospholipid metabolism	1%	0%	1%	1%
Membrane protein	**7%**	0%	**9%**	**10%**
Motility and Attachment	1%	0%	**5%**	0%
Nucleotide biosynthesis and metabolism	0%	0%	0%	0%
Putative enzymes	**7%**	0%	**7%**	**8%**
Secreted Factors (toxins, enzymes, alginate)	0%	0%	0%	0%
Transcriptional regulators	**7%**	0%	**11%**	**11%**
Translation, post-translational modification, degradation	0%	0%	1%	1%
Transport of small molecules	**7%**	0%	**9%**	**6%**
Two-component regulatory systems	1%	0%	4%	**5%**
Hypothetical, unclassified, unknown	52%	100%	24%	23%

*Percent of deleted genes per gene function are presented here. PseudoCAP functions are available on the Pseudomonas Genome Database (https://www.pseudomonas.com/). A convergent evolution by deletion was identified in a common region between A- and F- isolates. Percent of deleted genes per gene function are indicated by “A- and F- isolates.” When over than 5% of genes for a given PseudoCAP function were affected (except for “Hypothetical, unclassified, unknown” function), values were indicated in bold.*

Moreover, in A-isolates, the deleted region contained genes encoding EAL domain-containing proteins, which are involved in c-di-GMP degradation (a second messenger involved in biofilm formation), and genes involved in virulence (*hcnABC*, encoding an hydrogen cyanide synthase; the effector *exoY* of the type III secretion system; and *cupA1* to *cupA5*, involved in *fimbriae* synthesis).

### Phenotypic Analyses

Fitness and biofilm formation assays were performed for A-, D-, and F-isolates (*n* = 35 isolates) for which genomic deletions occurred over time.

**FIGURE 5:**
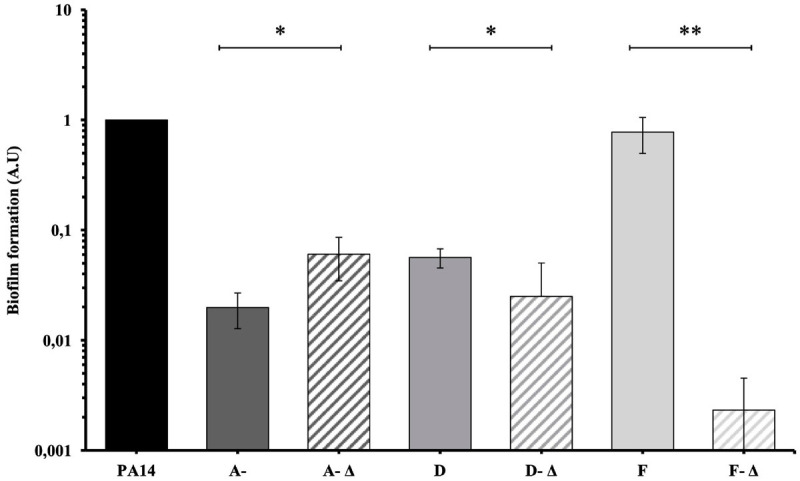
Mean biofilm formation of 35 *P. aeruginosa* urinary isolates collected from three patients (A, D, and F) compared to PA14. Biofilm formation was evaluated by crystal violet in microtiter plates and was compared between isolates with genomic deletions (-Δ) and without genomic deletion for each patients. A- Δ: A-III-3, and A-III-5 isolates; D- Δ: D-II-2 to D-II-5 isolates; F- Δ: F-II-1 to F-II-5 isolates. Significance was identified as *P* < 0.05 (*) or *P* < 0.01 (**) by Kruskal–Wallis test.

A- and D-isolates were low biofilm-producers (Figure 5). An increased biofilm formation was identified for A-Δ isolates compared to the others (*P* < 0.05). In contrast, the ability of the D-Δ isolates was reduced (*P* < 0.05). In parallel, early F-isolates were high biofilm-producers while deleted late F- isolates produced significantly less biofilm (*P* < 0.01).

**FIGURE 6:**
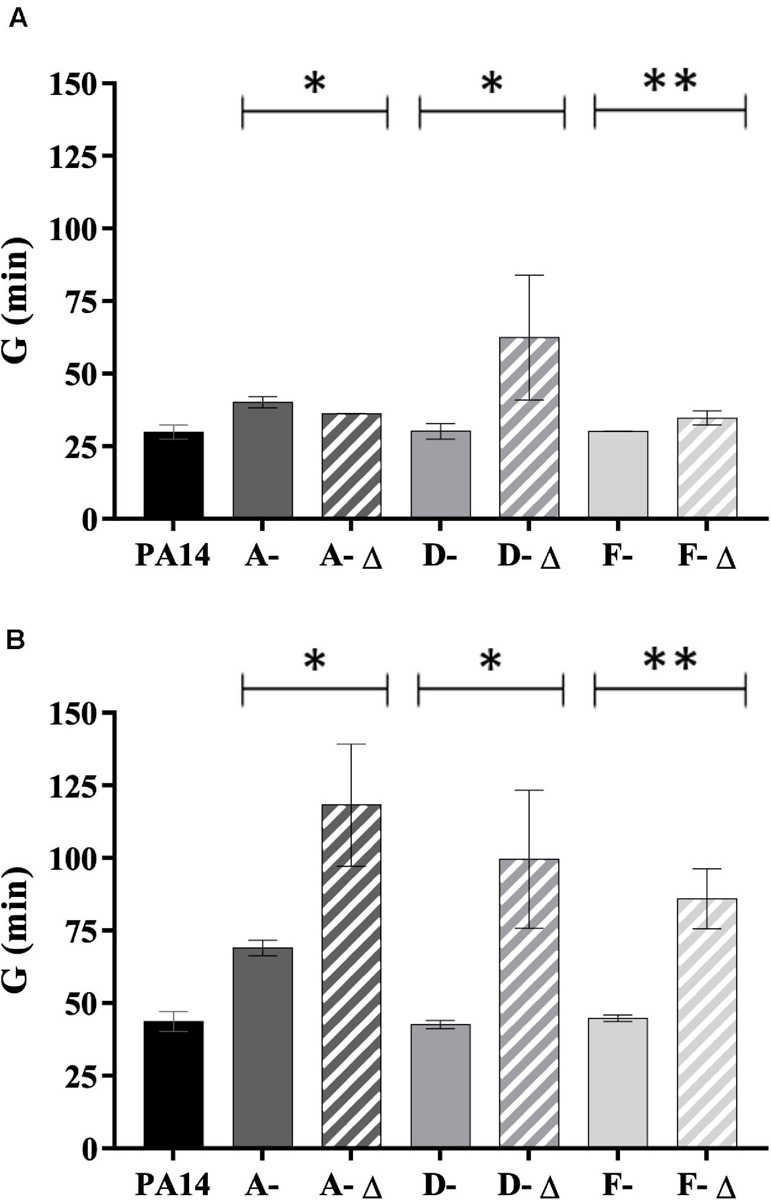
Generation time of 35 *P. aeruginosa* urinary isolates collected from three patients (A, D, and F) compared to PA14. Generation time (G) (min) in **(A)** TSB broth and **(B)** artificial urine medium of isolates with (-Δ) and without genomic deletion. A- Δ: A-III-3, and A-III-5 isolates; D- Δ: D-II-2 to D-II-5 isolates; F- Δ: F-II-1 to F-II-5 isolates. Significance was identified as *P* < 0.05 (*) or *P* < 0.01 (**) by Kruskal–Wallis test.

Isolates with a genomic deletion had a significant lower fitness in TSB broth (for D- and F-isolates) and in AUM (for isolates from all patients) (Figure 6). The reverse trend was observed for A-isolates in TSB broth (*P* < 0.05). Interestingly, the generation time ratio (defined as the mean generation time of isolates with genomic deletion on the mean generation time of isolates without one for a given patient) was near to 2 in AUM (1.7 for A-isolates, 2.3 for D-isolates, and 1.9 for F-isolates). Thus, isolates with genomic deletion grew about half as fast as isolates without a genomic deletion in AUM.

## Discussion

*Pseudomonas aeruginosa* is a major cause of healthcare associated UTI, characterized by frequent relapses (Djordjevic et al., 2013; Gomila et al., 2018). Numerous studies explored mechanisms of *P. aeruginosa* persistence during chronic pulmonary infections in CF patients (Caballero et al., 2015; Marvig et al., 2015b; Bianconi et al., 2018; Bartell et al., 2019), but little is known about its pathoadaptive adaptation in the urinary tract. To this end, up to five isolates of *P. aeruginosa* were collected in longitudinal urine samples from seven hospitalized patients.

All patients included in our study were colonized or infected over time by a single clone type, well-predicted by *in silico* MLST. Nevertheless, distinct sublineages have been identified in the second urine sample collected from one patient (D), suggesting a progressive replacement of the naive initial *P. aeruginosa* population over time. This is consistent with longitudinal studies of CF infections, which showed that chronic *P. aeruginosa* infections were often associated with diversification to distinct variants (Marvig et al., 2015b; Bianconi et al., 2018; Bartell et al., 2019). Even though our sampling period was shorter (488 days at most) than these studies (where inclusion could occurred nearly a decade apart), Bartell et al. (2019) pointed that rapid adaptation occur within 2–3 years timeline after colonization in CF patients.

To our knowledge, this is the first comprehensive whole genome analysis of within-host evolution of *P. aeruginosa* in the urinary tract. It has highlighted the molecular mechanisms of its adaptation, which was mostly driven by two distinct mechanisms: SNPs and large genomic deletions.

Single nucleotide polymorphisms analysis revealed that an average of less than 10 SNPs differentiated early from late isolates for a given patient. The number of SNPs identified was much lower than that detected for isolates collected during CF infections (up to 27,249 SNPs per strain in Bianconi et *al.*’s study) (Bianconi et al., 2018). This is probably because we used here the intra-patient reference sequence to limit the phylogenetic distance between isolates and the reference genome while CF studies usually use a reference sequence (*P. aeruginosa* DK2 or PAO1) (Marvig et al., 2015b; Bianconi et al., 2018). Nevertheless, we cannot exclude that the first urine sample of each patient included here did not correspond to the first episode of UTI or AB and therefore we could have underestimated within-host evolution.

However, in order to quantitatively compare evolution by SNPs over time, we have evaluated the number of SNPs per genome per year. For most patients, the mean number of SNPs per genome per year (ranging from 0 to 126) could have been higher than that previously described for chronic CF infections (1–3) (Marvig et al., 2015a). Interestingly, the highest rate observed for one patient (126, patient D) reached that of 89–106 identified for hypermutator strains (Marvig et al., 2015a). For this patient, no SNP was identified in DNA mismatch repair genes (*mutS* and *mutL*) that often characterize hypermutator strains (Colque et al., 2020), but two SNPs in genes involved in DNA replication, recombination, modification and repair were identified, as previously described (Qin et al., 2018). The hypermutable phenotype would be interesting to confirm *in vitro* in further analyses.

Late stage SNPs with moderate or high effect were identified in four patients, all from urine samples collected more than 100 days apart (B, D, F, G). In contrast to CF infections (Marvig et al., 2015b), no pathoadaptive genes that could suggest a convergent evolution were identified. This could be due to the heterogeneity of the patients included here, particularly in terms of age, catheterization, and urinary disorders, which could greatly influence the bacterial environment and *P. aeruginosa* within-host adaptation. Nevertheless, SNPs in genes encoding transcriptional regulators and two component systems were identified in three of the four patients, suggesting that remodeling of regulatory networks might be important in *P. aeruginosa* adaptation to the urinary tract. Similarly, 10 of the 52 pathoadaptive genes identified during a longitudinal study in CF infections encoded transcriptional regulators (Marvig et al., 2015b).

In addition to mutations, and similarly to Bianconi et al. (2018), we identified that bacterial persistence could be associated with loss events. Indeed, genomic deletions were identified in isolates collected from three of the seven patients studied (A, D, F) over up to 100 days apart. The size of the genomic deletions observed in our study (35–361 kbp) was slightly smaller than those previously reported during CF infections, which could reach up to 8% (525 kbp) of the genome (Rau et al., 2012).

Analysis of the deleted genes showed a convergent evolution on 70 CDS for two patients (A, F). Among them, deletion of the *hmgA* and *galU* genes have been previously reported *in vitro* (after meropenem exposure) or *in vivo* particularly during CF infections (Rau et al., 2012; Cabot et al., 2016; Hocquet et al., 2016; Wardell et al., 2019). Deletion of *hmgA* resulted in pyomelanin hyperproduction, which was also phenotypically observed for the isolates of our study that presented the deletion of this gene. The *galU* gene is involved in polysaccharide biosynthesis, and its deleted mutant was shown to be devoid of O-antigen, and promoted persistence of *P. aeruginosa* in chronic lung infection by immune evasion (Rodríguez-Rojas et al., 2009; Hocquet et al., 2016). Furthermore, as *galU* has previously been described as necessary for phage adsorption, its deletion led to a phage resistance mechanism (Le et al., 2014).

The convergent evolution in patients A and F led to the deletion of genes PA1965 to PA2034. Interestingly, this genomic area was previously described as subject to genomic deletion. In a study focused on six pyomelanin-producing mutants of *P. aeruginosa* (Hocquet et al., 2016), 46 genes in the vicinity of *hmgA* (from PA1974 to PA2019) were deleted in all the pyomelanin-producing isolates. Similarly, Wardell et al. (2019) identified large genomic deletions (from 225 kb up to 480 kb) that led to the loss of the PA2022 to PA2208 genes in 13 meropenem-resistant mutants. Thus, the deletion of genes in the vicinity of *hmgA* seems to provide an advantage for *P. aeruginosa* adaptation to human host. Gene function analysis identified that convergent evolution by deletion mostly affected genes involved in carbon compound catabolism, membrane proteins, and transcriptional regulators. Reduction in the catabolic repertoire was previously described in clinical CF isolates over time (La Rosa et al., 2019). In the same way, in an oxygen-limiting environment such as urinary tract, *P. aeruginosa* could divert its energy sources from sugar-derived carbon sources to denitrification or arginine fermentation (Tielen et al., 2013).

We then studied whether the large genomic deletions observed for some isolates had a phenotypic impact. We first studied biofilm formation, since chronic colonization is known to be associated with a switch from planktonic to sessile lifestyle and biofilm formation (Olivares et al., 2019). In two patients (A, D); who didn’t undergo urine catheterization, all isolates – whether they had the genomic deletion or not – were low-biofilm producers. These results were correlated with the observation made by Bartell et al. (2019) where biofilm formation didn’t significantly correlate with duration of colonization. Isolates with genomic deletion from the third patient (F), who had intermittent self-catheterization, produced significantly less biofilm than isolates without deletion. This phenotypic observation was probably not explained by the function of the deleted genes as none were involved in biofilm formation. However, the deleted isolates also presented SNPs in genes involved in alginate biosynthesis and regulation (*algG, algB*, and *mucA*), which could be involved in the reduction of biofilm production.

Next, we analyzed the fitness of our isolates in rich and artificial urine medium as several pathoadaptive genes (involved in central metabolism or antimicrobial resistance) have been shown to optimize pathogen fitness during CF infections (Marvig et al., 2015a). We showed that isolates with genomic deletion presented a lower fitness than isolates without deletion, particularly in AUM. These results are similar to those of Rodríguez-Rojas et al. (2009), who identified that isolates with genomic deletion presented a lower fitness (during a 48 h of co-infection with the wild-type strain in an acute respiratory model) that contributed to a decreased clearance and an increased persistence of *P. aeruginosa* in chronic lung infections. Further analyses based on continuous cultures are needed to get closer to the urinary dynamic state.

Numerous studies have showed that *P. aeruginosa* adaptation to pulmonary airway during chronic infections was associated with mutations in genes involved in antimicrobial, like those of the MexAB-OprM efflux pump (*mexA, mexB, nalD*) or genes involved in fluoroquinolones resistance (*gyrA, gyrB*) (Marvig et al., 2015b; López-Causapé et al., 2018; Bartell et al., 2019; Colque et al., 2020). In contrast, in our study, isolates from only one patient (D) harbored late stage SNP in a gene involved in antimicrobial resistance. This mutation in *mexS*, involved in regulation of the MexEF-OprN efflux pump, was correlated with increased within-sample diversity in terms of antimicrobial resistance profiles. In parallel, genomic deletions were associated for two patients (A, F) with the loss of the *mexX, mexY, mexZ* genes (involved in the MexXY-OprM efflux pump and aminoglycosides resistance) (López-Causapé et al., 2018), but they were correlated with phenotypic aminoglycoside-resistance for only one patient. Thus, acquisition of antimicrobial resistance doesn’t appear to provide a main advantage for *P. aeruginosa* adaptation in the urinary tract, even if a longer follow-up of the patients would have been useful to confirm this hypothesis. However, further genotypic analyses by real-time reverse transcription-PCR would be needed to determine the level of expression of genes potentially involved in antimicrobial resistance.

This study presented some limitations. First, a higher number of isolates per urine sample may be of interest to explore intra-host diversity and limit the risk of identifying late stage SNPs that may have been present in isolates from the first sample not analyzed in this study. Second, using two separate methodologies for genome sequencing may have biased the identification of the SNPs. Nevertheless, this approach allowed the mapping of the genomes on an intra-patient genome reference that was sequenced by a long-read technology. Furthermore, it could be interesting to explore other genomic adaptation mechanisms, such as short insertions-deletions. Third, genomic analyses required the use of numerous successive bioinformatics tools, each with its own sensitivity and specificity.

Finally, all patients included in our study were infected or colonized by a given clone type over time, even though diversification could occur. *P. aeruginosa* adaptation to the urinary tract frequently occurred by SNPs and/or deletion in genes encoding transcriptional regulators, two-component systems, and carbon compound catabolism. Limitation of oxygen and nutrients in the urinary tract could drive *P. aeruginosa* to adapt its metabolism in favor of non-carbon energy sources and then to remodel its regulatory networks. Further metabolomic and phenotypic studies (such as biofilm formation in artificial urine medium) would be of interest to identify the regulatory pathways used by *P. aeruginosa* for adaptation to urinary conditions. On the other hand, metagenomic analyses could be interesting to explore the dynamics and interplay of the urinary microbiome with *P. aeruginosa* persistent bacteriuria.

## Data Availability Statement

The datasets presented in this study can be found in online repositories. The names of the repository/repositories and accession number(s) can be found below: PRJNA656414.

## Ethics Statement

According to the French regulation on observational database analyses, this study did not need specific informed consent requirements; it was registered by the Directorate of Clinical Research and Innovation of the Rouen University Hospital Centre under the number 2018/413/OB.

## Author Contributions

AC, SD, MP-C, and EJ-B: conception and design. AC, MG, and TF: phenotypic experiments. AC and ML: genomic analyses. AC, ML, MG, FG, FA, and SL: interpretation of data. AC, ML, SD, and MP-C: manuscript preparation. All authors amended and approved the final version of the manuscript.

## Conflict of Interest

The authors declare that the research was conducted in the absence of any commercial or financial relationships that could be construed as a potential conflict of interest.

## Footnotes

1 https://www.eucast.org/

2 https://github.com/ncbi/pgap/

3 https://bitbucket.org/genomicepidemiology/kma.git

4 https://github.com/marieleoz/pairwise-SNPs-using-Snippy

5 https://cge.cbs.dtu.dk/services/PlasmidFinder/

6 https://www.pseudomonas.com/
