# Supplementary material for: Within-Host Microevolution of Pseudomonas aeruginosa Urinary Isolates: A Seven-Patient Longitudinal Genomic and Phenotypic Study
Source: Front Microbiol. 2021 Jan 14;11:611246. doi: 10.3389/fmicb.2020.611246 (PMC7840598; doi:10.3389/fmicb.2020.611246)

Supplementary Figure 1. Phylogenetic analysis of the 108 genomes included in the study, with a focus on the ST308 sequences.
A. The phylogenetic tree was constructed using Parsnp software, based on the assemblies of the 108 genomes studied. Sequence type (ST) defined by in silico MLST are indicated in the tree. Each color indicates the isolates collected from a given patient. Isolates from patients D and E, which belonged to the same ST (ST308), clustered together.
B. Illumina assembly genomes from patients D and E as well as from the five ST308 references available (AR_0353, B10W, Pa58, PASGNDM345, PASGNDM699) were submitted to phylogenetic analysis using Parsnp, with P. aeruginosa LESB58 full genome sequence as reference and outgroup (not shown to optimize the scale of the tree). Bootstrap values are indicated when >70.

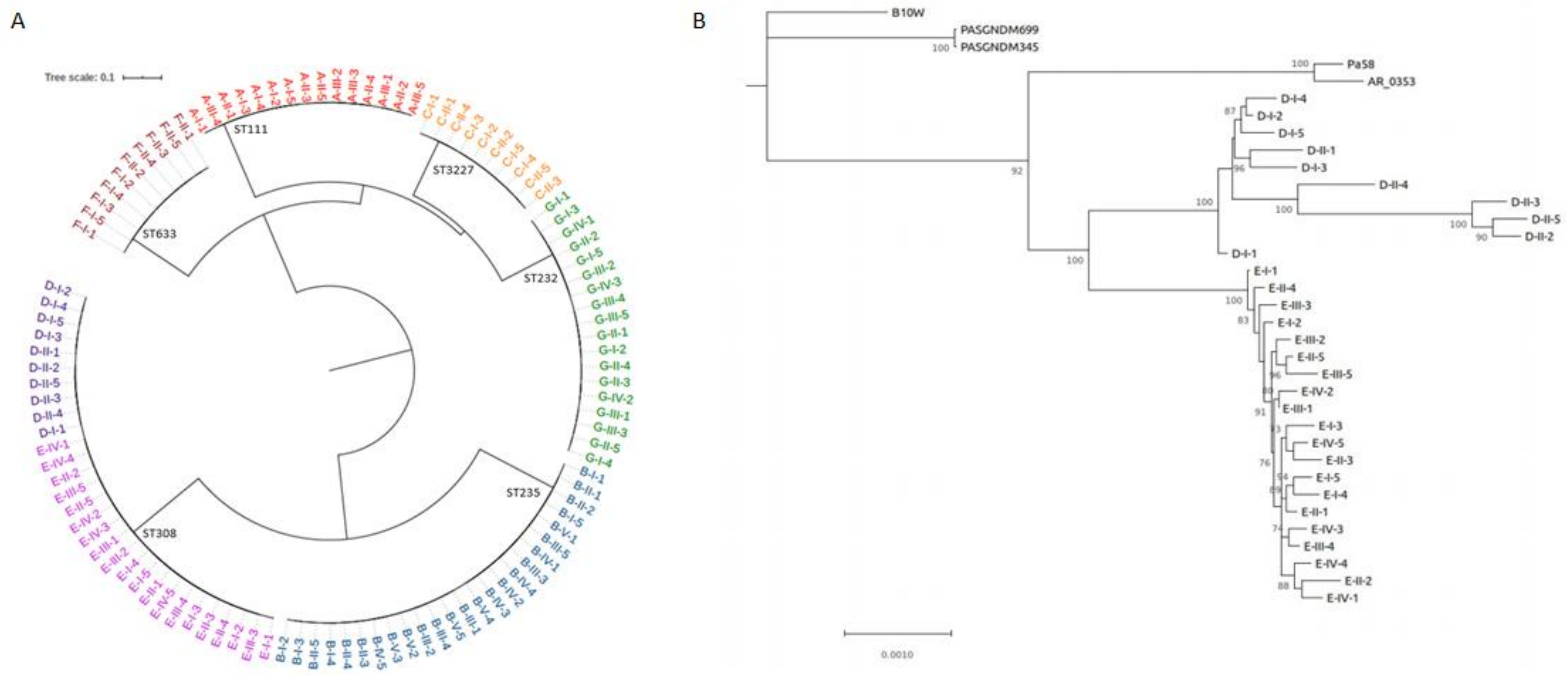

Supplement: Supplementary file 8 [file Image_1.PDF]
